# Comparison of Neutrophil Function in Granulocyte Concentrates From Prednisone- and G-CSF-Treated Donors: Effect of Stimulant, Leukapheresis and Storage

**DOI:** 10.3389/fmed.2022.839475

**Published:** 2022-03-04

**Authors:** Andréa Murru, Marie-Ève Allard, Guillaume Paré, Myriam Vaillancourt, Lucie Boyer, Marie-Pierre Cayer, Julien Vitry, Patricia Landry, Marie-Michèle Labrecque, Nancy Robitaille, Donald R. Branch, Mélissa Girard, Maria J. Fernandes

**Affiliations:** ^1^Infectious and Immune Diseases Division, CHU de Québec Research Center, Laval University, Québec, QC, Canada; ^2^Department of Microbiology-Infectious Diseases and Immunology, CHU de Québec Research Center, Faculty of Medicine, Laval University, Québec, QC, Canada; ^3^Medical Affairs and Innovation, Hema-Québec, Québec, QC, Canada; ^4^Transfusion Medicine, Hema-Québec, Québec, QC, Canada; ^5^Center for Innovation, Canadian Blood Services, Departments of Medicine and Lab Medicine and Pathobiology, University of Toronto, Toronto, ON, Canada

**Keywords:** granulocyte concentrates, neutrophils, leukapheresis, G-CSF, prednisone

## Abstract

Transfusion of granulocyte concentrates (GC) is an alternative therapy for neutropenic patients with life-threatening infections. While neutrophils are the main source of antimicrobial activity, only neutrophil numbers are used to certify GCs. The objective of this study was thus to functionally characterize neutrophils in GCs prepared by leukapheresis from G-CSF-stimulated donors and compare to the less characterized prednisone GCs. GCs prepared from healthy donors stimulated with prednisone and then G-CSF after a 6-month washout period were analyzed prior to and after leukapheresis, and after storage. Leukocyte composition, neutrophil viability, calcium mobilization, chemotaxis, phagocytosis, reactive oxygen species, cytokine production and metabolites were determined. G-CSF GCs contained significantly more neutrophils than prednisone GCs of which 40% were immature. In comparison to non-stimulated healthy donor neutrophils, prednisone GC neutrophils exhibited enhanced phagocytosis and G-CSF GC neutrophils showed decreased chemotaxis but increased IL-8 production. Leukapheresis altered prednisone GC neutrophil responses. Storage had a significant, negative impact on G-CSF GC neutrophils compared to prednisone GC neutrophils. G-CSF and prednisone GC neutrophils thus differ in maturity and function, and G-CSF GC neutrophils are more sensitive to storage. Functional testing of GC neutrophils and better storage conditions would improve the quality of this blood product.

## Introduction

Life-threatening infections are a major health concern due to growing resistance to antimicrobial and antifungal therapies (1). This is of particular concern for neutropenic patients as they are highly susceptible to infections due to a low number of neutrophils (2), the primary source of anti-microbial defenses (3–5). Moreover, the prevalence of neutropenic patients with antimicrobial resistant infections is increasing due to the growing use of aggressive chemotherapy and hematopoietic stem cell transplants (6). A potential lifesaving therapy for these patients is the transfusion of granulocytes.

Granulocyte transfusions (GTXs) temporarily increase neutrophil count until the bone marrow restores granulopoiesis (7). To harvest the minimal number of granulocytes for GTX (10^10^/transfusion) healthy donors are stimulated with G-CSF and/or a corticosteroid. In Canada, all GCs are prepared from prednisone-stimulated donors by leukapheresis, whereas in the US GC donors are stimulated with G-CSF (8). In Europe, buffy-coat GCs are more routinely used.

Neutrophil anti-microbial activity encompasses several effector functions including chemotaxis, phagocytosis and pathogen destruction by antimicrobial peptides and reactive oxygen species (ROS). In addition, neutrophils sequester and destroy pathogens by releasing extracellular traps (NETs) composed of chromatin, histones and intracellular proteins (9, 10). Cytokines also play a role by further activating neutrophils and neighboring cells, and promoting the additional leukocyte recruitment of neutrophils and monocytes (11–13). Without these defenses, survival from an infection is limited to a few days in persons with absolute neutropenia (14–16).

While GCs are transfused to temporarily provide antimicrobial defenses, only the absolute neutrophil count (ANC) is used to certify GCs for transfusion (17). This is problematic as studies report functional differences between GCs and non-stimulated healthy donor neutrophils. Impaired phagocytosis was observed in G-CSF GC neutrophils (18) and elevated pro-inflammatory cytokine levels in GC supernatants, a likely cause of febrile reactions (19, 20). In contrast, other studies observed no differences in chemotactic activity (21, 22) or ROS production (22) in G-CSF GC neutrophils compared to neutrophils of non-stimulated healthy donors. Together, these findings underscore the need for a comprehensive characterization of GC neutrophils to ensure their optimal antimicrobial capacity prior to transfusion. Since GC neutrophils prepared from prednisone-stimulated donors are less well characterized and currently used for GTX, the objective of this study was to compare the functional responses of prednisone and G-CSF GC neutrophils prior to and after leukapheresis and during storage.

## Materials and Methods

### Recruitment and GC Collection

Ten healthy donors were recruited by Héma-Québec for two GC donations (the first after prednisone stimulation and the second after G-CSF stimulation) separated by a 6-months wash out period prior to leukapheresis with SpectraOptia (Terumo) according to Héma-Québec guidelines. All donations were collected in blood collection bags containing sodium citrate (46.7%) and hydroxyethyl starch (HESPAN^®^ 6% B. Braun Medical Inc.) to enhance red blood cell sedimentation at a ratio of 13:1 (product:anticoagulant solution). GCs were sent by the Globule Laval collection center to Québec City Héma-Québec for irradiation at 25 Gy prior to delivery to our laboratory for analysis on the day of collection (D1) as well as 24 h (D2) and 48 h (D3) post-collection. GCs were stored at room temperature without agitation. A pre-leukapheresis blood sample was drawn from donors in acid-citrate-dextrose (ACD) (BD Vacutainer) tubes. Blood sample and GC composition was determined at the Héma-Québec collection center with a cell counter (Ac•T 5diff hematology analyser, Beckman Coulter). One donation was excluded from the functional analysis because of a change in the sedimentation agent during the first GC donation. For comparison, blood donations from unstimulated frequency matched healthy donors was drawn in ACD tubes by the Clinical Research Platform at the CHU de Québec-Laval University and were used as control.

### Material and Reagents

HES was purchased from Braun Medical Inc. (US). Fura-2- acetoxymethyl ester (Fura-2AM), CM-H2DCFDA, calcein-AM, the 7AAD viability staining solution and the pHRodo Red Zymosan A Bioparticles kit were obtained from Thermo Fisher (ON, Canada); dextran T500, cytochrome C, Hemacolor ® Rapid staining kit from Sigma-Aldrich (ON, Canada); lymphocyte separation medium, RPMI-1640, BSA (bovin serum albumin) and FBS (fetal bovine serum) from Wisent Bioproducts (QC, Canada); and the ChemoTx microplates (101–8) from NeuroProbe (MD, USA).

### Neutrophils and Peripheral Blood Mononuclear Cell Isolation

Pre-leukapheresis circulating neutrophils were isolated by density gradient from peripheral blood at room temperature under sterile conditions as described in Fernandes et al. (23). Neutrophils were resuspended in Mg^2+^-free HBSS containing 1.6 mM CaCl_2_ at 10 x 10^6^ ml^−1^. Neutrophils isolated from GCs did not require dextran sedimentation as they already contain hydroxyethyl starch, a sedimenting agent. The mononuclear cell layer was harvested after density gradient separation, washed and resuspended in PBS at 10 x 10^6^ ml^−1^.

### Flow Cytometry Analysis

A LSRII flow cytometer was used for immunophenotyping with a twelve antibody panel and for fluorescent non-opsonized zymosan phagocytosis analysis. A CantoII flow cytometer was used for viability and intracellular ROS production assays. Cells were always used at 10 x 10^6^ ml^−1^, stained at room temperature and kept on ice until analysis. All assays are explained in detail in the Supplementary Material.

## Results

### GC Collection

Ten healthy donors that met the exclusion and inclusion criteria in Table 1 were recruited to donate GCs. Inter-donor variability was minimized by harvesting from all donors a prednisone GC followed by a G-CSF GC with a wash out period of 6 months between GC donations. The average blood volume filtrated from prednisone-stimulated donors during leukapheresis was 4,964 ml (4211-6001 ml) and 4,831 ml (3,611–6,039 ml) for G-CSF-stimulated donors (Table 2). Ten healthy, age and sex frequency-matched donors were also recruited for comparative purposes.

**Table 1:** Donor characteristics and collection regimens.

	**Apheresis group (*****n*** **=** **10)**		**Non-stimulated control group (*n* = 10)**
Sex (M:F)	M:5 F:5		M:5 F:5
Age, median year (range)	42 (23–58)		42 (21–61)
GC final volume (ml)	350		–
	**Pred**	**G-CSF**	
Leukapheresis duration, min (range)	96 (64–138)	80 (66–104)	–
Dose	50 mg (*per os*)	300 mcg (intravenous)	–
Time before apheresis	12 to 18 h	24 h	–
	Inclusion criteria Age 18–60 Men and women		
	Exclusion criteria Infectious diseases transmitted by blood (hepatitis B and C, syphilis, HIV 1 and 2 and HTLV I/I) Complications during apheresis procedure Recent infections that required treatments (antibiotics, antifungals)		

**Table 2:** Composition of donor peripheral blood and granulocyte concentrates.

	**Prednisone (*n* = 9)**	**G-CSF (*n* = 10)**
**Donors peripheral blood (pre-collection)**		
Total leukocytes (x10^9^/L) (%)	8.42	24.56
Neutrophils	6.30 (74.8)	20.55 (83.7)
Lymphocytes	1.52 (18.1)	2.01 (8.2)
Monocytes	0.49 (5.1)	1.16 (4.7)
Eosinophils	0.08 (1.0)	0.49 (2.0)
Basophils	0.03 (0.4)	0.35 (1.4)
**Granulocyte concentrate**		
Filtrated blood volume (ml)	4,964	4,832
Total leukocyte (x10^9^/L) (%)	62.85 (100)	109.45 (100)
Neutrophils	35.70 (56.8)	82.12 (75.0)^**^
Lymphocytes	18.29 (29.1)	17.89 (16.3)
Monocytes	6.59 (10.5)	7.35 (6.7)
Eosinophils	0.75 (1.2)	1.58 (1.4)
Basophils	1.52 (2.4)	0.52 (0.5)
Dose ANC/GC unit (x10^10^)	1.28	2.85
Hemoglobin (g/L)	34.56	22.20^**^
Hematocrit (L/L)	0.108	0.071^**^
Platelets (x10^9^/L)	297.6	261.0

*ANC, absolute neutrophil count. Comparison of prednisone vs. G-CSF group: t-test*,

* *p-value < 0.05*;

** *p-value < 0.01*.

### Comparison of Neutrophil Counts in Prednisone and G-CSF GCs

**Figure 1:**
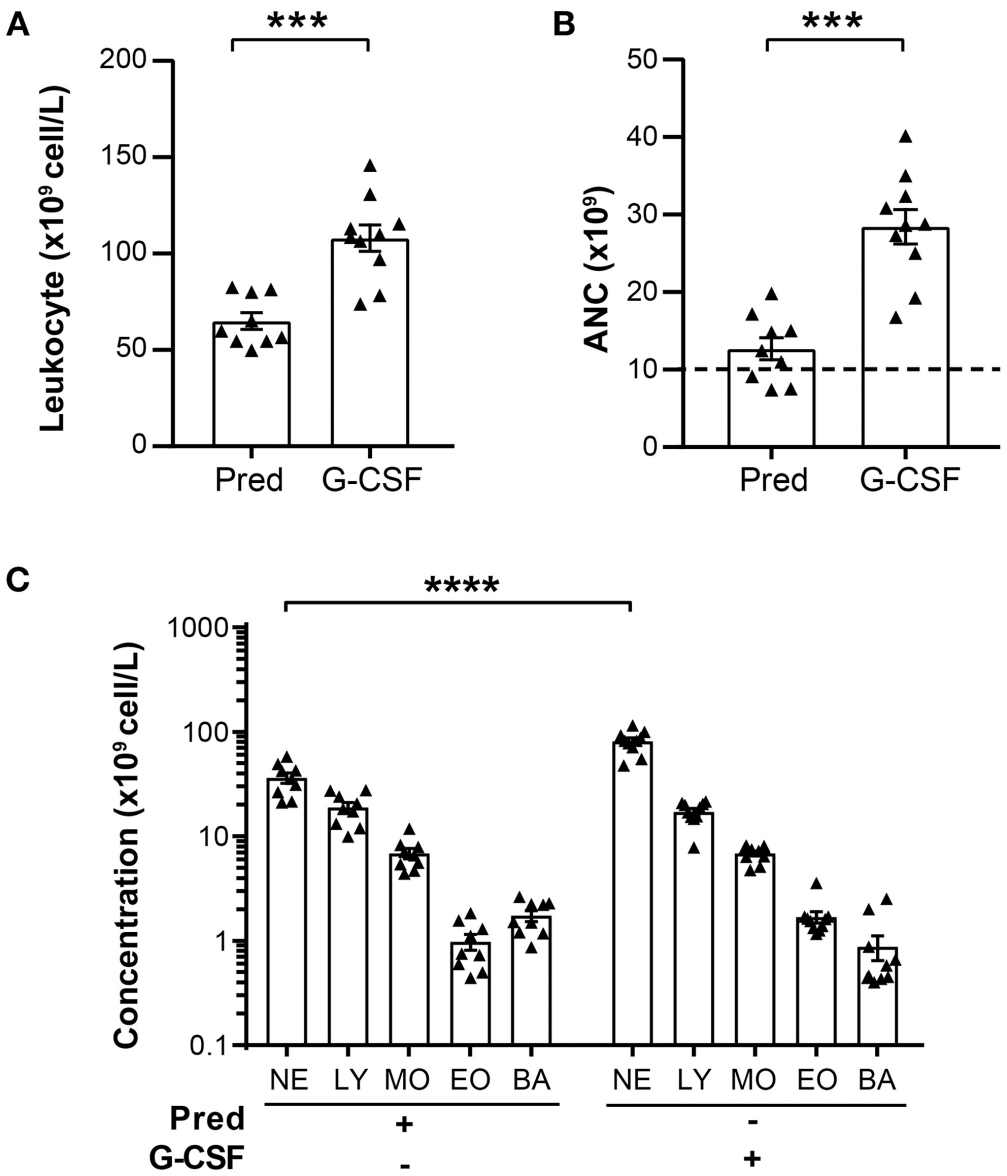
Leukocyte composition in prednisone and G-CSF GCs. **(A)** Total leukocyte concentration in prednisone and G-CSF-derived GCs. **(B)** ANC calculated for a final GC volume of 350 ml. The dashed line indicates the minimal number of neutrophils (10^10^) required for granulocyte transfusion. **(C)** Concentration of leukocyte populations (neutrophils, NE; lymphocytes, LY; monocyte, MO; eosinophils, EO; basophils, BA) in prednisone and G-CSF GCs expressed as cell number per liter. Each dot represents an individual GC donor. Statistical analysis: Mann-Whitney test performed to compare leukocyte concentration and ANC between prednisone and G-CSF GCs: ***p-value < 0.001. Sidak multiple comparisons test performed to compare the concentration of leukocyte populations between prednisone and G-CSF GCs: ****p-value < 0.0001. These data are compiled from the analysis of 9 prednisone and 10 G-CSF GCs.

Since G-CSF mobilizes neutrophils more efficiently than glucocorticoids, we analyzed the leukocyte content in both types of GCs. Prednisone stimulation consistently generated GCs of a similar or significantly lower leukocyte concentration than G-CSF (62.9 x 10^9^.L^−1^ and 109.5 x 10^9^.L^−1^ leukocytes, respectively; Table 2, Figure 1A). The minimal ANC required *per* transfusion of 10^10^ neutrophils was thus observed in 6/9 (67%) of prednisone GCs compared to 10/10 G-CSF GCs (Figure 1). Neutrophils were 1.5 to 4-fold more abundant in G-CSF than prednisone GCs and comprised an average of 75.5% of all leukocytes in G-CSF GCs compared to 56.8% in prednisone GCs.

While neutrophils are the most abundant cells in GCs, other leukocytes are also present in this cellular product. Notably, lymphocytes represented 30% of all leukocytes in prednisone GCs and 16% in G-CSF GCs (Table 2). A substantial number of monocytes are also detected, 11% in prednisone GCs and 7% in G-CSF GCs. Minor leukocytes populations in these GCs included basophils and eosinophils. Together, these findings indicate that G-CSF is more efficient at mobilizing neutrophils for GCs than prednisone, and that GCs contain a significant proportion of lymphocytes and monocytes in addition to neutrophils.

### Viability and Surface Marker Expression of Prednisone and G-CSF Mobilized Neutrophils

**Figure 2:**
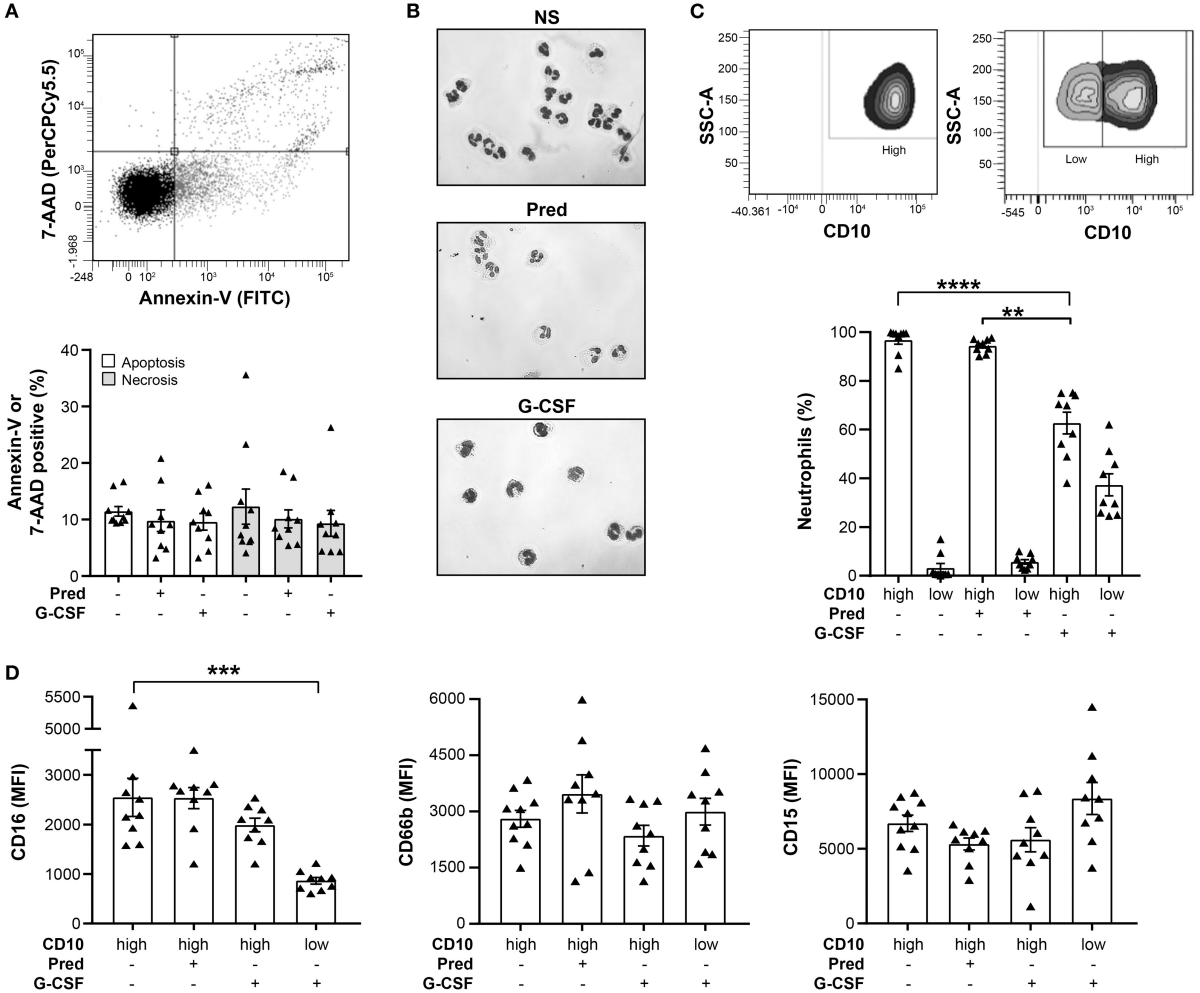
Viability, morphology, and cell-surface marker expression of prednisone- and G-CSF-mobilized neutrophils. **(A)** The proportion of neutrophil, spontaneous apoptosis and necrosis was determined by 7-AAD/Annexin-V staining in prednisone and G-CSF GCs as well as neutrophils of non-stimulated, healthy donors. A representative two-dimensional plot of Annexin-V/7-AAD staining is shown *above* the graph. **(B)** Nuclear morphology of neutrophils from control, non-stimulated-donors **(NS)** *(top)*, prednisone- *(middle)* and G-CSF-mobilized neutrophils *(bottom)* after Pappenheim staining. **(C)** Proportion of circulating neutrophils that display high or low CD10 cell-surface expression determined by flow cytometry. A representative plot of CD10 staining in a CD10^high^ *(left)* and a CD10^high^/CD10^low^ *(right)* sample is shown *above* the graph. **(D)** Cell-surface expression of CD16 *(left)*, CD66b *(middle)* and CD15 *(right)* determined by flow cytometry and expressed as mean fluorescence intensity **(MFI)**. Statistical analysis: Dunn's multiple comparison test was performed to compare cell-surface marker expression between prednisone- and G-CSF-mobilized neutrophils, **p-value < 0.01; ***p-value < 0.001; ****p-value < 0.0001. These data are compiled from the analysis of 9 prednisone and 9 G-CSF GCs as well as neutrophils of 10 non-stimulated healthy donors.

To characterize the effect of prednisone and G-CSF stimulation on neutrophils *in vivo*, we compared their viability, maturity and cell-surface marker expression prior to leukapheresis. Spontaneous apoptosis and necrosis was not significantly altered in G-CSF GC neutrophils compared to neutrophils of non-stimulated healthy donors (Figure 2A).

Since G-CSF induces the release of immature neutrophils from the bone marrow (24), we stained G-CSF mobilized neutrophils with the maturity marker CD10. G-CSF induced the release of both mature (60% of CD10^high^) and immature (40% of CD10^low^) neutrophils into the circulation (Figures 2C,D). The presence of neutrophils with a mature or immature nuclear morphology confirmed this observation (Figure 2B). Contrary to low-density, immature neutrophils that increase in numbers in some disease states, G-CSF GC immature neutrophils have a similar density as mature neutrophils as they pellet to the bottom of the density gradient. In contrast, prednisone only mobilized CD10^high^ neutrophils with multi-lobular nuclei, a typical feature of mature neutrophils (Figures 2B,C) (25). An additional distinctive feature of G-CSF GC immature neutrophils is their significantly lower expression of CD16 (CD16^low^) compared to their CD16^high^ mature counterparts. The expression of the other cell-surface markers tested in prednisone and G-CSF-mobilized neutrophils was not altered (Figure 2D). Together, these data indicate that prednisone and G-CSF mobilized neutrophils had similar viability but differed in their maturation stage.

### Neutrophil Antimicrobial Defenses in Prednisone and G-CSF GCs

**Figure 3:**
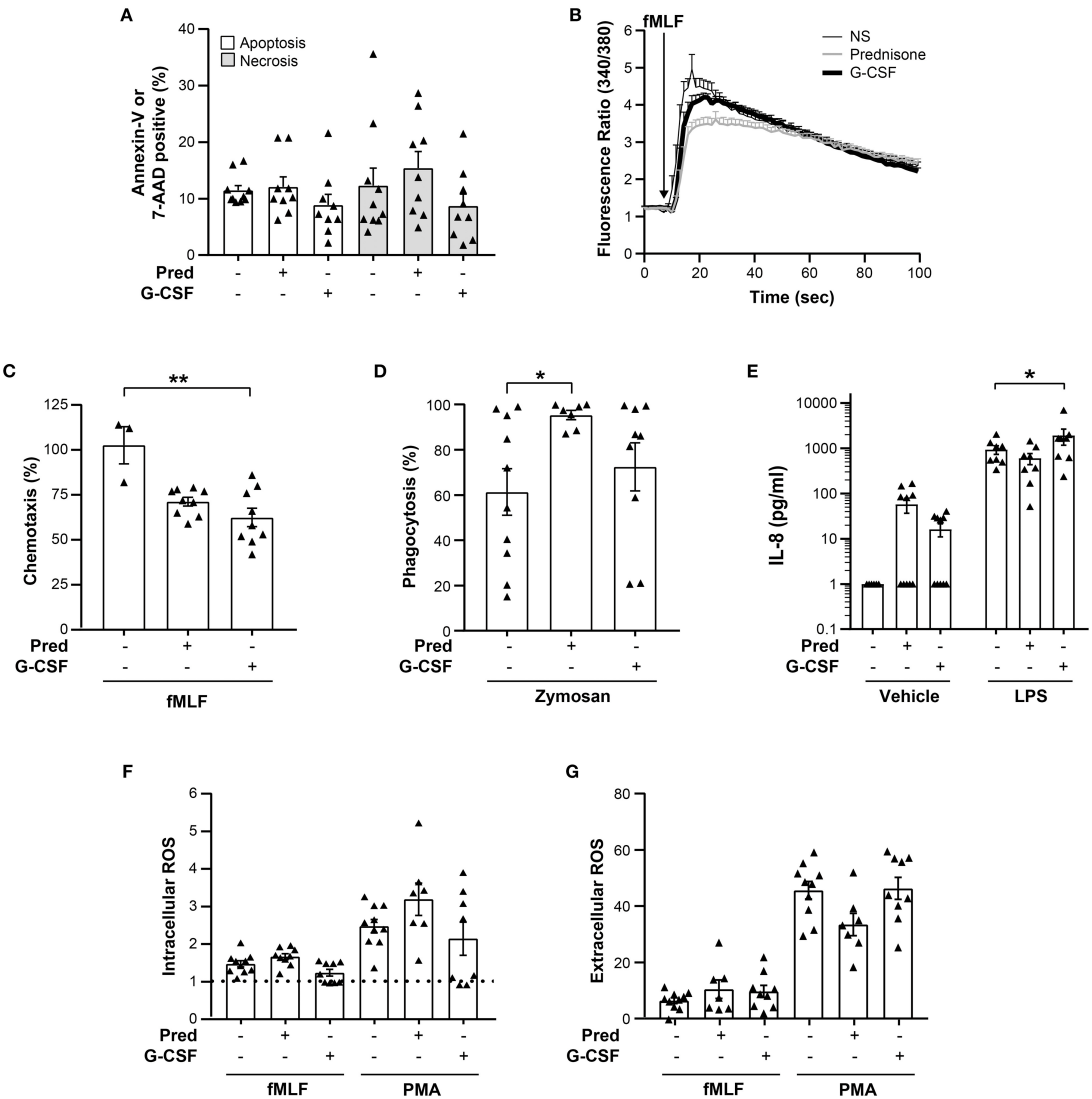
Viability and function of prednisone and G-CSF GC neutrophils. **(A)** Proportion of apoptosis and necrosis determined by Annexin-V and 7-AAD staining of neutrophils isolated from non-stimulated, healthy donors *(-/-)*, and prednisone or G-CSF GCs after leukapheresis on the day of collection. **(B)** Intracellular calcium levels measured with a spectrofluorometer after fMLF stimulation as in “Materials and Methods.” The kinetics of the change in intracellular calcium concentration is shown. For **(A,B)**, neutrophils of 10 non-stimulated, healthy donors, 9 prednisone and 9 G-CSF GCs were analyzed. **(C)** Chemotaxis toward fMLF expressed as the maximal proportion of migrated neutrophils [non-stimulated, healthy donors (*n* = 3), prednisone (*n* = 9) and G-CSF GC donors (*n* = 9)]. **(D)** Phagocytosis of non-opsonized pHRodo Red zymosan conjugated bioparticles is expressed as the proportion of phagocytic neutrophils [non-stimulated, healthy donors (*n* = 10), prednisone (*n* = 7) and G-CSF GC donors (*n* = 9)]. **(E)** LPS-induced IL-8 release by neutrophils determined by ELISA (n=8 for all experimental conditions). **(F)** Intracellular ROS production induced by fMLF [non-stimulated, healthy donors (*n* = 10), prednisone and G-CSF GC neutrophils (*n* = 9)] or PMA [non-stimulated. healthy donors (*n* = 10), prednisone (*n* = 7) and G-CSF GC neutrophils (*n* = 8)]. Data are expressed as a MFI ratio of stimulated cells/cells incubated in DMSO (diluent). A ratio of 1 corresponds to the absence of an increase in ROS production compared to the DMSO-treated neutrophils *(dotted line)*. **(G)** Extracellular ROS production measured with cytochrome *c* by neutrophils incubated with DMSO, fMLF [non-stimulated, healthy donors (*n* = 10), prednisone GCs (*n* = 7), G-CSF GCs (*n* = 9)] or PMA [non-stimulated, healthy donors (*n* = 10), prednisone (*n* = 7), G-CSF (*n* = 8)] expressed as the concentration of superoxide produced. Statistical analysis: Dunn's and Dunnett's multiple comparison test performed to compare neutrophils from non-stimulated, healthy donors with prednisone and G-CSF GC neutrophils, *p-value < 0.05; **p-value < 0.01.

Since neutrophils are the main source of antimicrobial activity in GCs, we assayed the key neutrophil functions required to fight infections in GC neutrophils 6 h post-leukapheresis. For comparative purposes, the assays were also performed on neutrophils from unstimulated, healthy donors. Of these neutrophil responses, the function that was significantly altered in prednisone GC neutrophils was the phagocytosis of non-opsonized zymosan. While considerable variability in the phagocytosis was observed amongst non-stimulated healthy donors, prednisone leveled this response to 90% phagocytosis in all donors (Figure 3). A diminution in fMLF-induced chemotaxis was also observed but did not reach significance. Likewise, no significant difference in the LPS-induced production of IL-8 and the fMLF and PMA-induced ROS production, or increase in cytoplasmic calcium was observed between prednisone or G-CSF GC and non-stimulated, healthy donor neutrophils (Figure 3).

G-CSF GC neutrophils also exhibited functional differences with non-stimulated, healthy donor neutrophils albeit for different functions (Figure 3). Chemotaxis induced by fMLF was significantly downregulated in G-CSF GC neutrophils whereas LPS-induced production of IL-8 was significantly enhanced (Figure 3). No significant differences in the other functional assays were observed. Together, these data reveal a mobilizing agent-dependent alteration in GC neutrophil function.

### Effect of Leukapheresis on the Function of Prednisone and G-CSF GC Neutrophils

**Figure 4:**
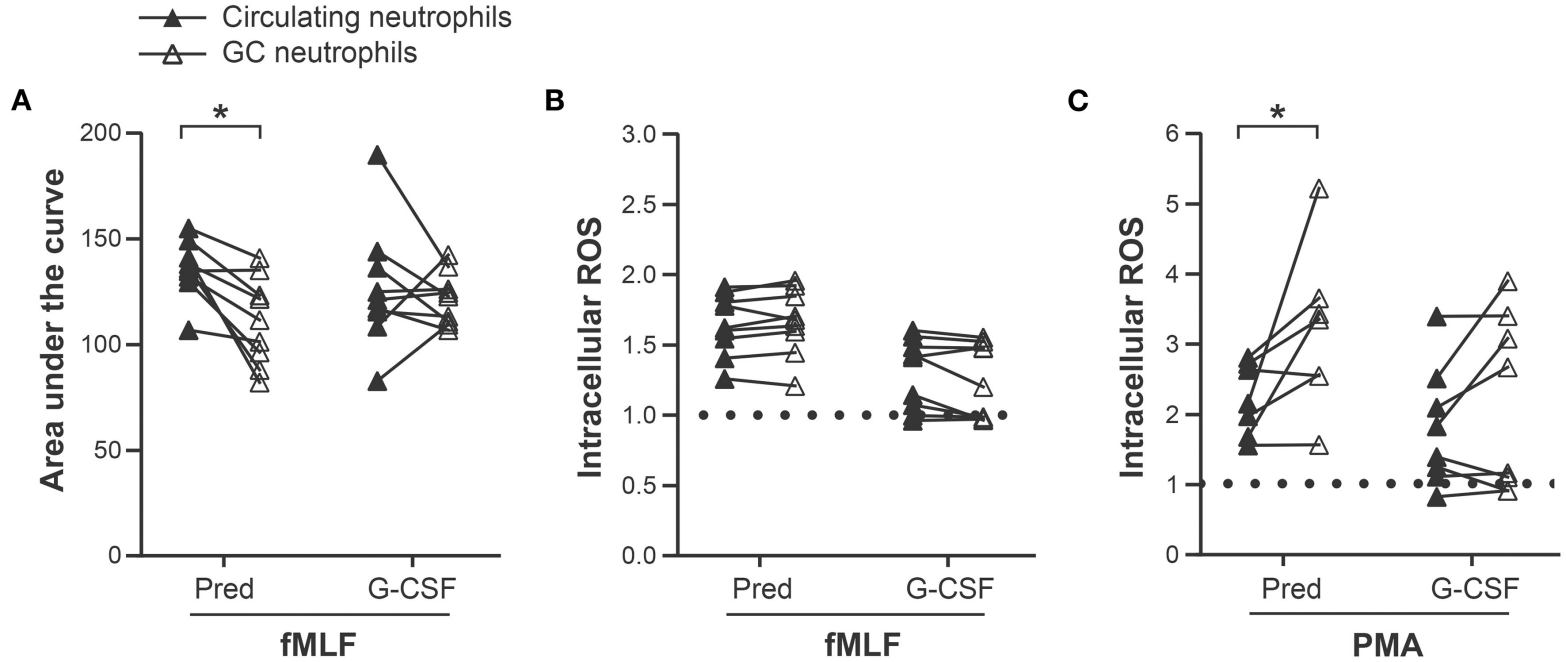
Effect of leukapheresis on neutrophil responses. **(A)** Intracellular calcium levels measured with a spectrofluorometer after fMLF stimulation of circulating neutrophils (pre-leukapheresis) and prednisone and G-CSF GC neutrophils (post-leukapheresis) as described in “Materials and Methods.” **(B)** fMLF and **(C)** PMA-induced intracellular ROS production measured with CM- H2DCFDA in circulating neutrophils and prednisone and G-CSF GC neutrophils. The results are expressed as a MFI ratio of stimulated cells/cells incubated in DMSO (diluent). The dotted line represents the absence of increase in ROS production (ratio = 1). Statistical analysis: Sidak multiple comparison test performed to compare circulating neutrophils with GC neutrophils from prednisone- and G-CSF-pre-treated donors: *p-value < 0.05. These data are compiled from the analysis of 9 donors for the fMLF stimulation and 7 prednisone and 8 G-CSF GCs for the PMA stimulation.

To determine whether leukapheresis contributes to the functional alterations observed in GC neutrophils, their responses were compared before and after leukapheresis. A significant diminution in the fMLF-induced increase in cytoplasmic calcium (Figure 4A) and an increase in PMA, but not fMLF, -induced intracellular ROS production in prednisone GC neutrophils was observed after leukapheresis (Figures 4B,C). In contrast, G-CSF GC neutrophils were unaffected by this procedure (Supplementary Figure 1). Together, these data indicate that prednisone GC neutrophils are more affected by leukapheresis than G-CSF GC neutrophils.

### Effect of Storage on Neutrophil Viability, Cell-Surface Markers, pH, and Metabolite Concentration

**Figure 5:**
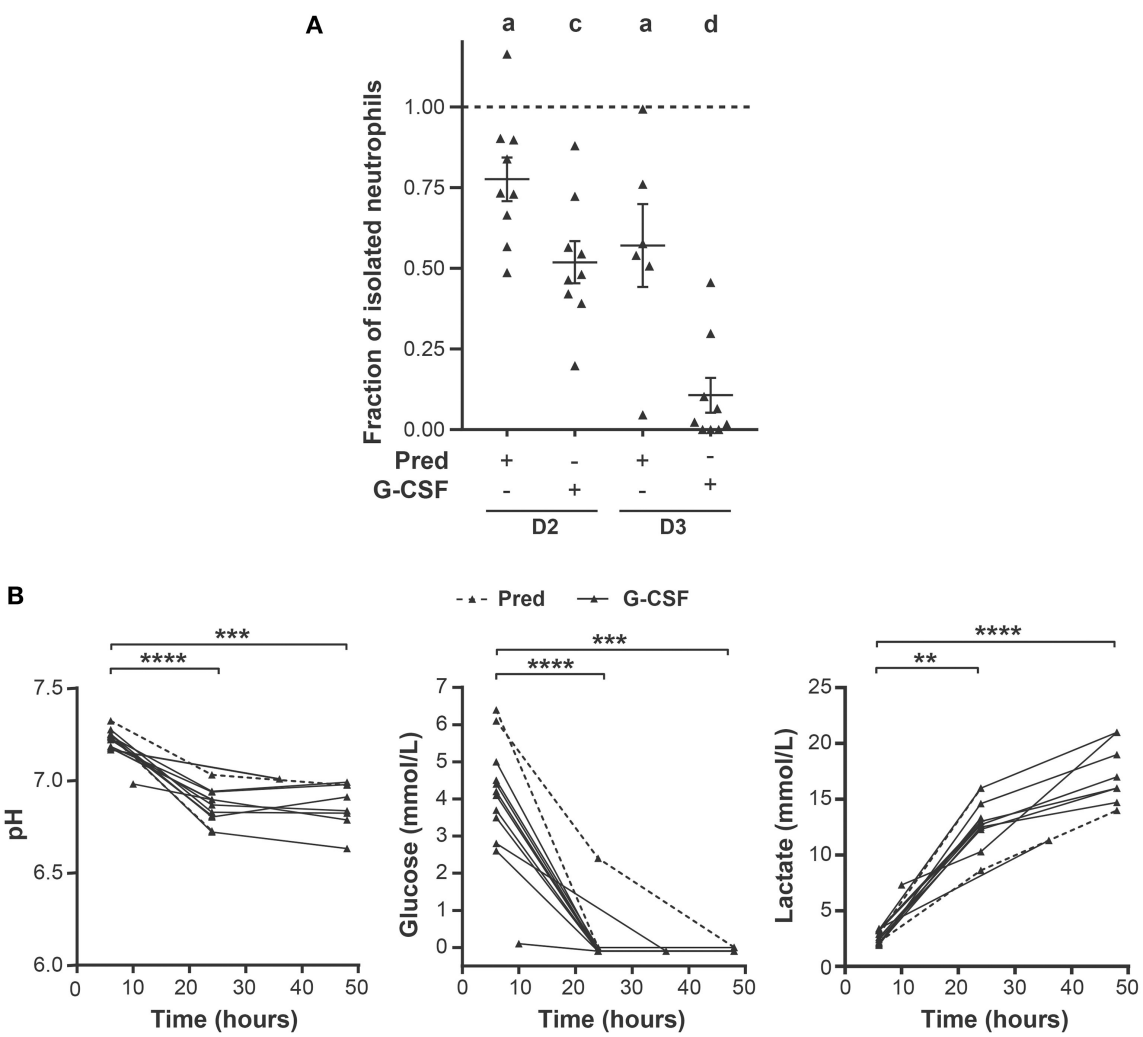
Effect of storage on neutrophil isolation from GCs and GC metabolite composition. **(A)** Ratio of the number of neutrophils isolated with a density gradient 24 h (D2) or 48 h (D3) post-leukapheresis, and the number of neutrophils isolated from GCs right after leukapheresis (D1); (D2/D1 and D3/D1). Sample size: D1 (*n* = 9), D2 (*n* = 9), D3 (*n* = 7). Cell counting was performed with a hemacytometer after trypan blue staining. The dashed line corresponds to a ratio of 1. **(B)** The pH *(left)*, glucose *(middle)* and lactate *(right)* concentrations were measured in GCs after the indicated times of storage. Prednisone, *dashed line* (*n* = 2), G-CSF *solid line*, (*n* = 10). Statistical analysis: A sample *t* test with a theorical mean of 1 was performed in **(A)**, ^a^*p*-value < 0.05; ^c^*p*-value < 0.001 and ^d^*p*-value < 0.0001. Sidak multiple comparison test was performed to compare GC metabolite content at 24 or 48 h of storage with that after 6 h of storage in **(B)**, **p-value < 0.01; ***p-value < 0.001; ****p-value < 0.0001.

**Figure 6:**
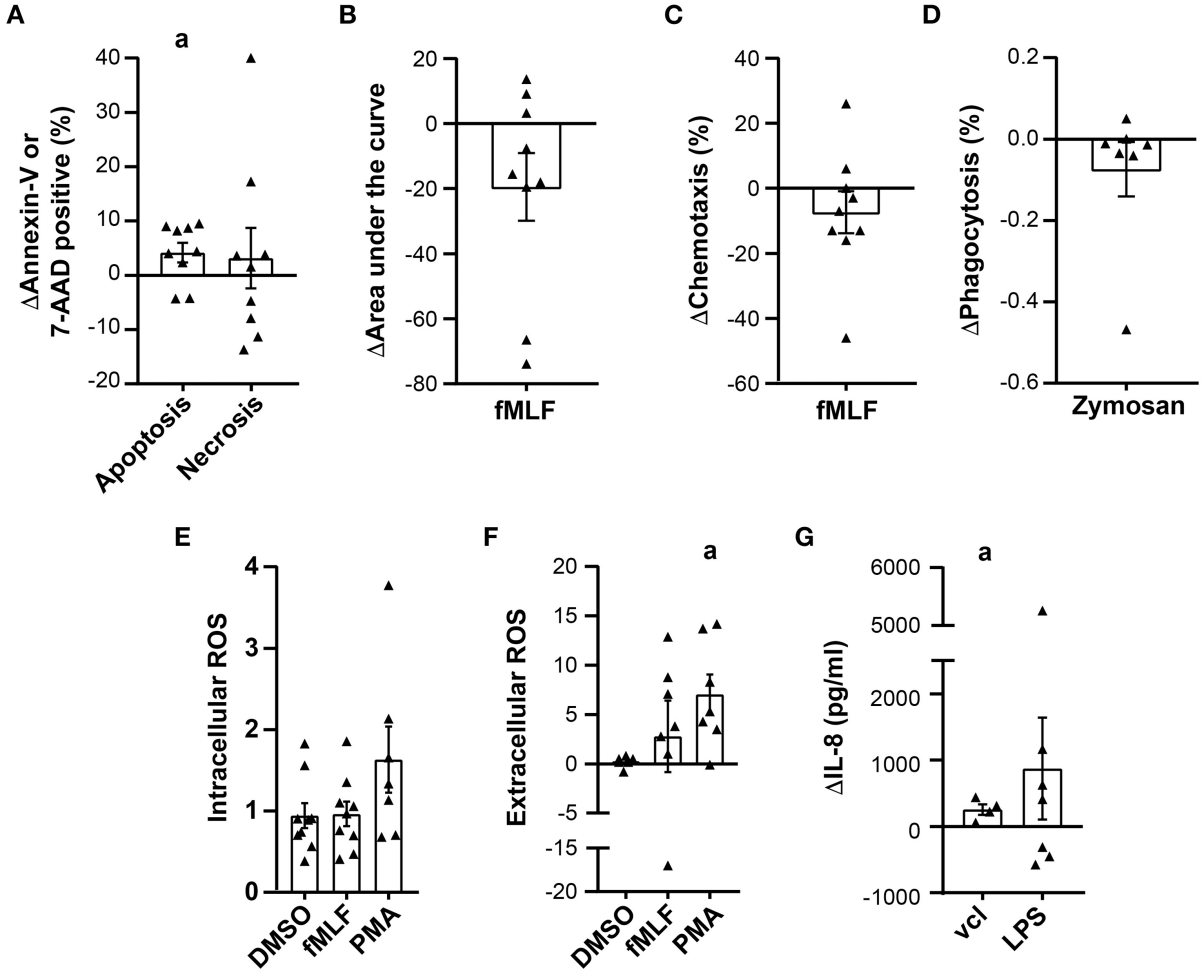
Variation in prednisone GC neutrophil viability and function after 24 h of storage. Viability and functional assay data are presented as the difference between the values obtained on the day of leukapheresis (D1) and 24 h post-leukapheresis (D2), (D2-D1), in all graphs except in **(E)**. **(A)** Difference in the proportion of apoptotic and necrotic neutrophils determined by Annexin-V and 7-AAD staining, respesctively (*n* = 9). **(B)** Difference in the fMLF-induced increase in intracellular calcium expressed as the area under the curve (*n* = 9). **(C)** Difference in the maximal proportion of neutrophils that migrated across a chemotaxis chamber toward fMLF (*n* = 9). **(D)** Effect of storage on the proportion of neutrophils that phagocytosed non-opsonized pHRodo Red Zymosan conjugated particles (*n* = 7). **(E)** Neutrophil fMLF and PMA-induced intracellular ROS production expressed as a ratio of the MFI (D1/D2); *n* = 9 and *n* = 7, respectively. **(F)** Change in fMLF and PMA-induced superoxide anion production expressed as the difference in O^2−^ produced on D1 and D2 (*n* = 7). **(G)** Difference of IL-8 production expressed in pg/ml (*n* = 8). Statistical analysis: One sample t test with a hypothetical mean of 0 [in **(A–D,F,G)**] or 1 (in **(E)** was performed to determine the significance of the difference or ratio between values obtained on D1 and D2, ^a^*p*-value < 0.05.

While the negative effect of storage on G-CSF GC neutrophil viability and function was reported in several studies (18, 26, 27), the impact of storage on prednisone GC neutrophils remains unknown. Storage negatively affects neutrophils as we were not able to isolate the predicted quantity of neutrophils from these GCs based on the neutrophil concentration in the GC prior to isolation. The number of neutrophils isolated from prednisone GCs after 24 and 48 h of storage declined by 20 and 40%, respectively, compared to the neutrophil yield on the day of collection (Figure 5A). Apoptosis of the isolated neutrophils harvested from prednisone GCs after 24 h of storage significantly increased by 8–10% in 4/9 donors (Figure 6A). As for cell-surface marker expression, CD66b expression significantly increased after 48 h of storage suggesting an increased degranulation (Supplementary Figure 2).

As observed for prednisone GC neutrophils, G-CSF GC neutrophils were also affected by storage. The most striking effect was the difficulty in isolating neutrophils from these GCs. The neutrophil yield dropped by 40% after 24 h storage and 80%, 48 h post-leukapheresis (Figure 5A). The functional data on these GCs is thus shown as supplementary data (Supplementary Figures 3, 4). Storage had no significant effect on cell-surface marker expression of G-CSF GC neutrophils *(data not shown)*.

A change in metabolite concentration and/or pH could explain the observed negative effects of storage on GCs. Data for two prednisone GC donations and for all G-CSF GCs was generated as the metabolite measurements began later in the study. We consistently observed a decrease in pH (7,25 to 6,82) 24 h post-collection in G-CSF GCs as well as glucose with a concomitant 5 to 8-fold increase in lactate concentration during storage (Figure 5B). Together, these data indicate that the decrease in GC neutrophil viability is highly likely due to rapid changes in pH and a significant change in metabolite concentration.

### Effect of Storage on Neutrophil Function

A diminution in most neutrophil responses including calcium mobilization, fMLF-induced chemotaxis and the phagocytosis of non-opsonized zymosan by prednisone GC neutrophils was observed after 24 h storage but did not reach significance (Figures 6B–D). In contrast, a significant increase was observed in both PMA-induced superoxide production and spontaneous release of IL-8 but not in intracellular ROS production (Figures 6E–G). The increase in superoxide production is suggestive of neutrophil priming during storage. Since the number of neutrophils that could be isolated from the collection bag diminished considerably with storage, functional data on prednisone GC neutrophils after 48 h storage and G-CSF GC neutrophils after 24 and 48 h storage may not be representative of all neutrophils in GCs (Supplementary Figures 3, 4). Together, these data reveal that G-CSF GC neutrophils rapidly deteriorate with storage and that storage differentially affects prednisone GC neutrophil effector functions.

## Discussion

Comparison of neutrophil functional studies performed on GCs prepared by different blood centers is challenging due to differences in GC preparation and analysis. Moreover, there are few reports on prednisone GCs. To address these gaps in our knowledge of GCs, we compared prednisone and G-CSF GC neutrophil viability and function in a single center study and on the same donors to minimize differences in GC production and inter-donor variability. Major differences in ANC, neutrophil maturity, functional capacity and response to storage were observed between prednisone and G-CSF GCs. To our knowledge, this is the first comprehensive study that compares the antimicrobial defenses of prednisone- and G-CSF GC neutrophils on the day of collection and during storage.

Mobilizing a sufficient number of neutrophils is considered crucial for the therapeutic efficacy of GTX. We show that G-CSF was more efficient at mobilizing neutrophils into circulation in all donors than prednisone corroborating a similar observation by Hiemstra et al. (28). Worel et al. (29) also reported that a lower proportion of prednisolone GCs (56%) contained the minimal ANC *per* GC unit for transfusion (29). These findings underscore the importance of verifying ANC prior to transfusion, especially for prednisone GCs due to its variable efficacy in mobilizing neutrophils.

A striking difference between prednisone- and G-CSF- GC neutrophils was the presence of 30–40% immature neutrophils in the latter. These neutrophils are not low-density granulocytes as they are of a higher density than PBMCs. The release of immature, low-density neutrophils from the bone marrow seems to require more than one injection of G-CSF as Marini et al. (30) reported their presence in the circulation in donors stimulated with G-CSF for 5 days (10 μg/kg/day). The difference in maturity between prednisone and G-CSF GC neutrophils is thus highly likely due to the preferential mobilization of neutrophils from the marginated pool by prednisone and bone marrow by G-CSF (31).

Functional analysis revealed major differences between GC and non-stimulated healthy donor neutrophils. Most notably, prednisone GC neutrophils exhibited a significant increase in non-opsonized zymosan phagocytosis. Whether this increased phagocytic capacity compensates for the lower ANC to preserve the overall antimicrobial efficacy in these GCs remains unknown. In contrast, G-CSF GC neutrophils migrated less efficiently than unstimulated donor neutrophils. This may be, in part, due to their incomplete differentiation as chemotaxis is one of the last functions to develop in mature neutrophils (32). Whether reduced chemotaxis is compensated *in vivo* by the higher ANC of these GCs and/or their increase in IL-8 production remains unknown and underscores a gap in our knowledge about functional compensation in neutrophils.

Comparison of GC neutrophil functional data between studies is challenging due to different study designs. Nevertheless, a comparison with the few reports resembling our study revealed that neutrophil function varies between GCs prepared by different blood centers The significant decrease in fMLF-induced chemotaxis in G-CSF GCs was also observed by Leavey (26) in GC neutrophils prepared from donors administered a higher dose of G-CSF for 5 consecutive days. In contrast, other studies (22, 33) reported increased basal chemotactic activity in neutrophils mobilized by a combination of G-CSF and dexamethasone (DEX) (22, 33). As for ROS production, Mochizuki (19) also demonstrated that ROS production was preserved in neutrophils of G-CSF and G-CSF/DEX GCs. In contrast to our findings, Joos found an increase in *E.coli* and fMLF-induced ROS in G-CSF GC neutrophils (34) underscoring the unmet need in GTX to certify GCs for transfusion with a neutrophil functional, quality control test. The only consistent finding irrespective of GC preparation was an increase in cytokine levels during storage including IL-8, IL-6, IL-1β and TNF-α (18, 19, 21, 27). Activated neutrophils may be a source of these cytokines as we observed for prednisone GC neutrophils.

Another source of variability between GCs stems from the inherent functional variability between healthy donors (35, 36). Neither prednisone nor G-CSF diminished GC neutrophil, interdonor functional variability. The only exception was prednisone's ability to increase the phagocytic capacity of GC neutrophils of all donors to the same level. While the molecular mechanism(s) involved in this phenomenon remain unknown, we postulate that prednisone increases the expression of pattern recognition receptors. How this increase in phagocytosis modifies the antimicrobial competency of prednisone GC neutrophils remains unknown.

Neutrophils are fragile cells *ex-vivo*. Even though glucocorticoids and G-CSF increase neutrophil viability (37, 38), GC neutrophil viability was not increased in stimulated donors on the day of collection or during storage. Neutrophil viability decreased significantly during storage most likely due to the rapid decrease in glucose and pH. The high leukocyte concentration in GCs and metabolic activity of red blood cells are likely causes of the pH decrease. Enriching the GC medium is thus crucial to preserve neutrophil viability and function (39). Several reports already demonstrated the possibility to extend neutrophil viability *ex vivo* with hypothermic solutions or the addition of anti-apoptotic agents and/or G-CSF (27, 39, 40).

In conclusion, the variability in neutrophil function between GCs underscores the unmet need of establishing a functional test to certify GCs for their antimicrobial properties before GTX and for GC donor selection. In addition, optimization of GC storage conditions will extend neutrophil viability *in vitro*, improve GC efficacy and expand its use in transfusion medicine.

## Data Availability Statement

The original contributions presented in the study are included in the article/Supplementary Material, further inquiries can be directed to the corresponding author/s.

## Ethics Statement

The study was performed according to the Declaration of Helsinki for studies with human subjects and approved by the CHU de Québec-Université Laval research Ethics Committee (# 2019-4493) and by the Héma-Québec research Ethics Committee (# 2018-012). Written informed consent was obtained from all the participants.

## Author Contributions

AM: significant contribution to performing the research, data compilation and analysis, participated in writing the manuscript, and prepared the figures. M-ÈA, GP, MV, LB, M-PC, JV, PL, and M-ML: all these co-authors contributed to performing the research and data compilation and part of the data analysis. NR: contributed to the data analysis and manuscript review. DB: contributed to the conceptualization, data analysis and manuscript review as well as funding acquisition. MG: contributed to the supervision of the personnel, conceptualization and methodology of the study, data analysis, manuscript review, and funding acquisition. MF: funding acquisition, resources and project administration, major contribution to the conceptualization and methodology of the study, the manuscript writing as well as review, editing, visualization, and supervised the personnel. All authors contributed to the article and approved the submitted version.

## Funding

This research was supported by funding from Héma-Québec and the Canadian Blood Services awarded to MF, DB, and MG. A MITACS/ Héma-Québec scholarship was awarded to AM (IT5795).

## Conflict of Interest

The authors declare that the research was conducted in the absence of any commercial or financial relationships that could be construed as a potential conflict of interest.

## Publisher's Note

All claims expressed in this article are solely those of the authors and do not necessarily represent those of their affiliated organizations, or those of the publisher, the editors and the reviewers. Any product that may be evaluated in this article, or claim that may be made by its manufacturer, is not guaranteed or endorsed by the publisher.
