# Supplementary material for: Comparison of Neutrophil Function in Granulocyte Concentrates From Prednisone- and G-CSF-Treated Donors: Effect of Stimulant, Leukapheresis and Storage
Source: Front Med (Lausanne). 2022 Mar 4;9:839475. doi: 10.3389/fmed.2022.839475 (PMC8934424; doi:10.3389/fmed.2022.839475)
Supplement: Supplementary file 1 [file Data_Sheet_1.pdf]

Supplementary Material

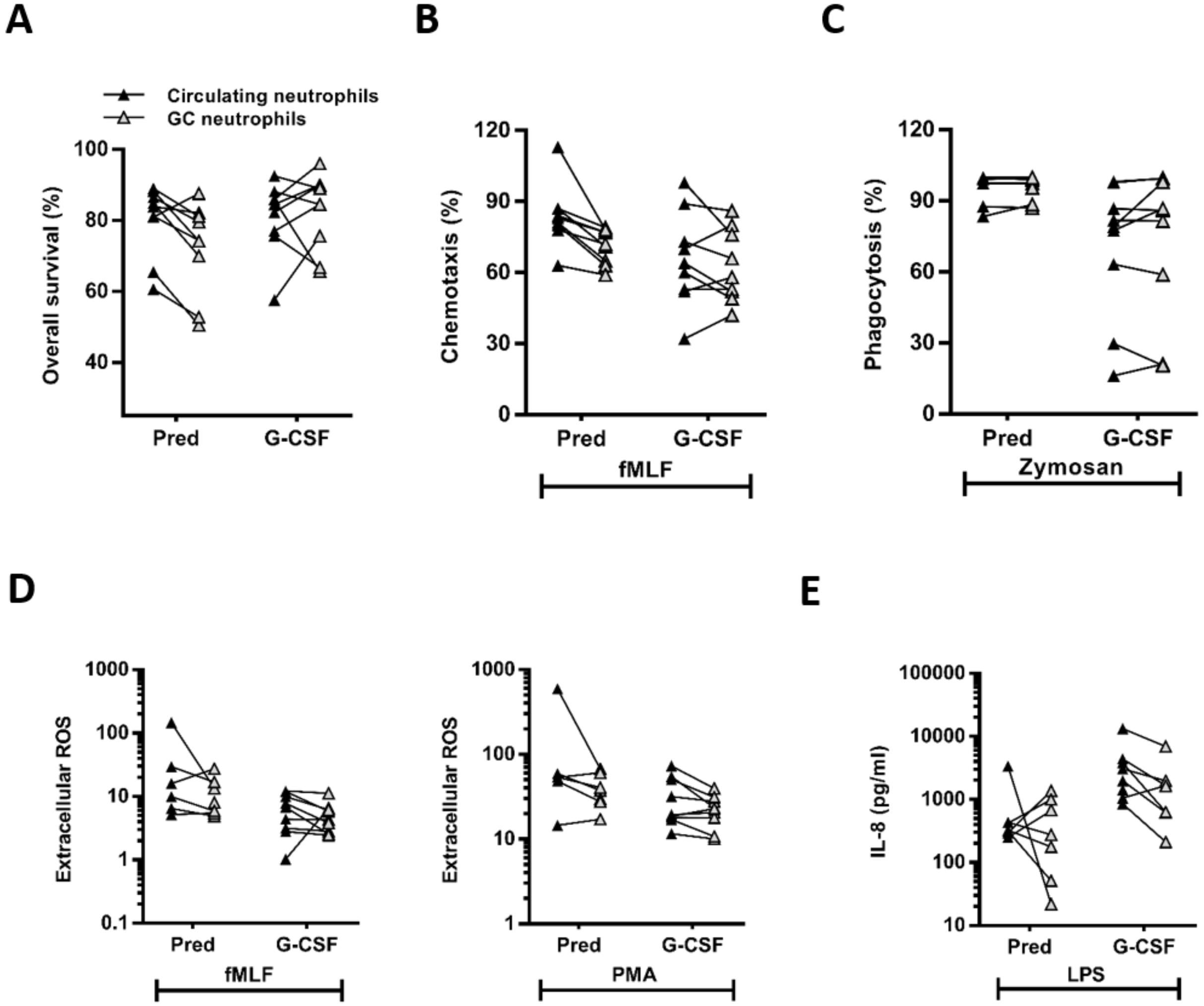

Fig S1: Leukapheresis has no significant effect on neutrophil viability and function. Viability and functional assays were performed on circulating (pre-leukapheresis) and GC (4 to 6 hrs post-leukapheresis) neutrophils of the same donors. *(A)* Overall survival of neutrophils was determined by Annexin-V/7-AAD staining (n=9). *(B)* Chemotaxis towards fMLF was measured in the ChemoTx disposable system and expressed as the maximal proportion of migrated neutrophils (n=9). *(C)* Proportion of neutrophils that phagocytosed non-opsonized pHRodo Red Zymosan conjugated bioparticles was determined by flow cytometry (prednisone (n= 7), G-CSF (n=8)). *(D)* Extracellular ROS production was measured with cytochrome c as in 'Materials and Methods' in neutrophils stimulated with fMLF *(left)* or PMA *(right)* (prednisone (n=6), G-CSF (n=9)) and expressed as the concentration of superoxide produced. *(E)* LPS-induced release of IL-8 by neutrophils was determined by ELISA (prednisone (n=8), G-CSF (n=9)). Data is expressed as the concentration of IL-8 produced after LPS stimulation subtracted from the basal IL-8 production in the presence of diluent. Statistical analysis: Sidak

multiple comparison test was performed to compare circulating neutrophils with GC neutrophils from prednisone- and G-CSF-pre-treated donors.

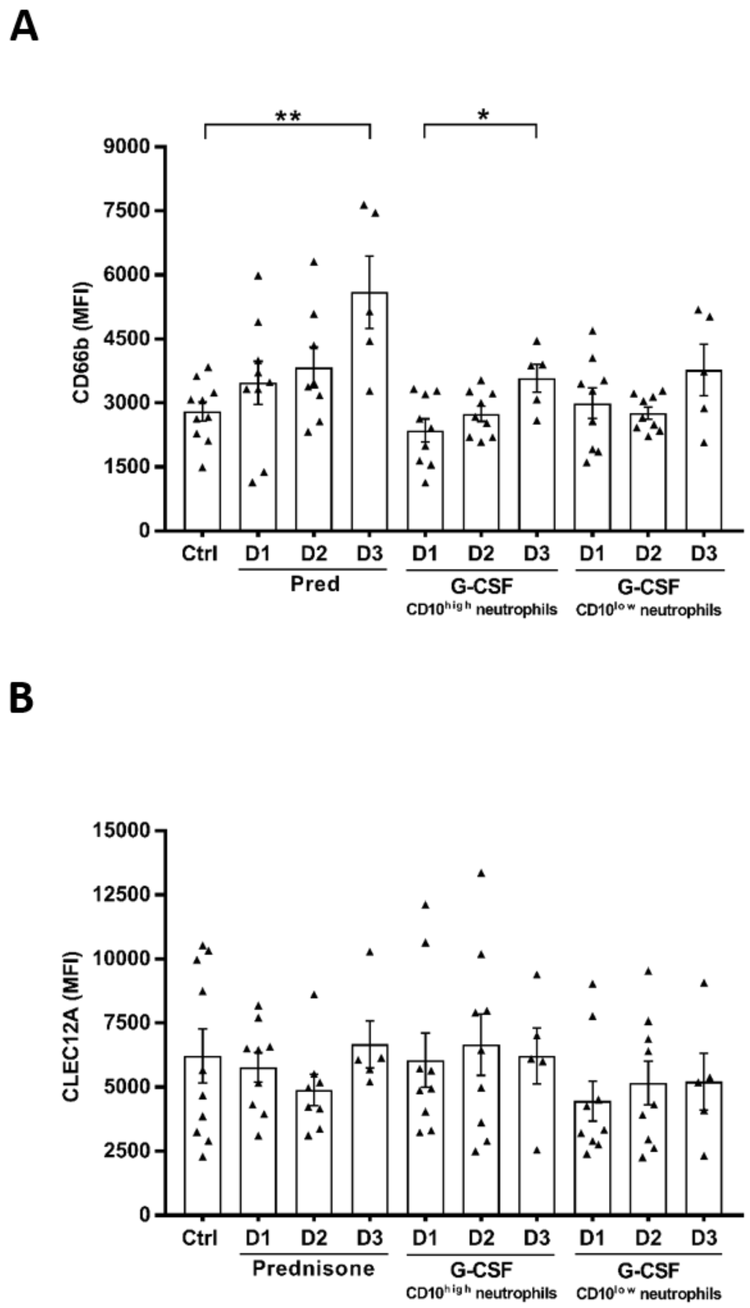

Fig S2: Change in neutrophil cell-surface marker expression during storage. *(A)* Cell-surface expression of CD66b was determined by flow cytometry on non-stimulated neutrophils of healthy donors *(Ctrl)* and prednisone and G-CSF GC neutrophils. Data are expressed as mean fluorescence intensity (MFI) at D1, D2 and D3 (D1: the day of leukapheresis; D2: 24 hrs and D3 48 hrs post-leukapheresis). *(B)* Cell-surface expression of CLEC12A was determined by flow cytometry as in *(A)*. These data are compiled from the analysis of 10 non-stimulated, healthy donors, 9 GC donors at D1, 8 prednisone- and 9 G-CSF-derived GCs at D2, 5 prednisone- and 6 G-CSF-derived GCs at D3. Statistical analysis: One way ANOVA with Dunn's multiple comparison test was performed to

compare cell-surface marker expression between non-stimulated, healthy donor neutrophils with prednisone and G-CSF GC neutrophils: *p-value < 0.05. **p-value < 0.01

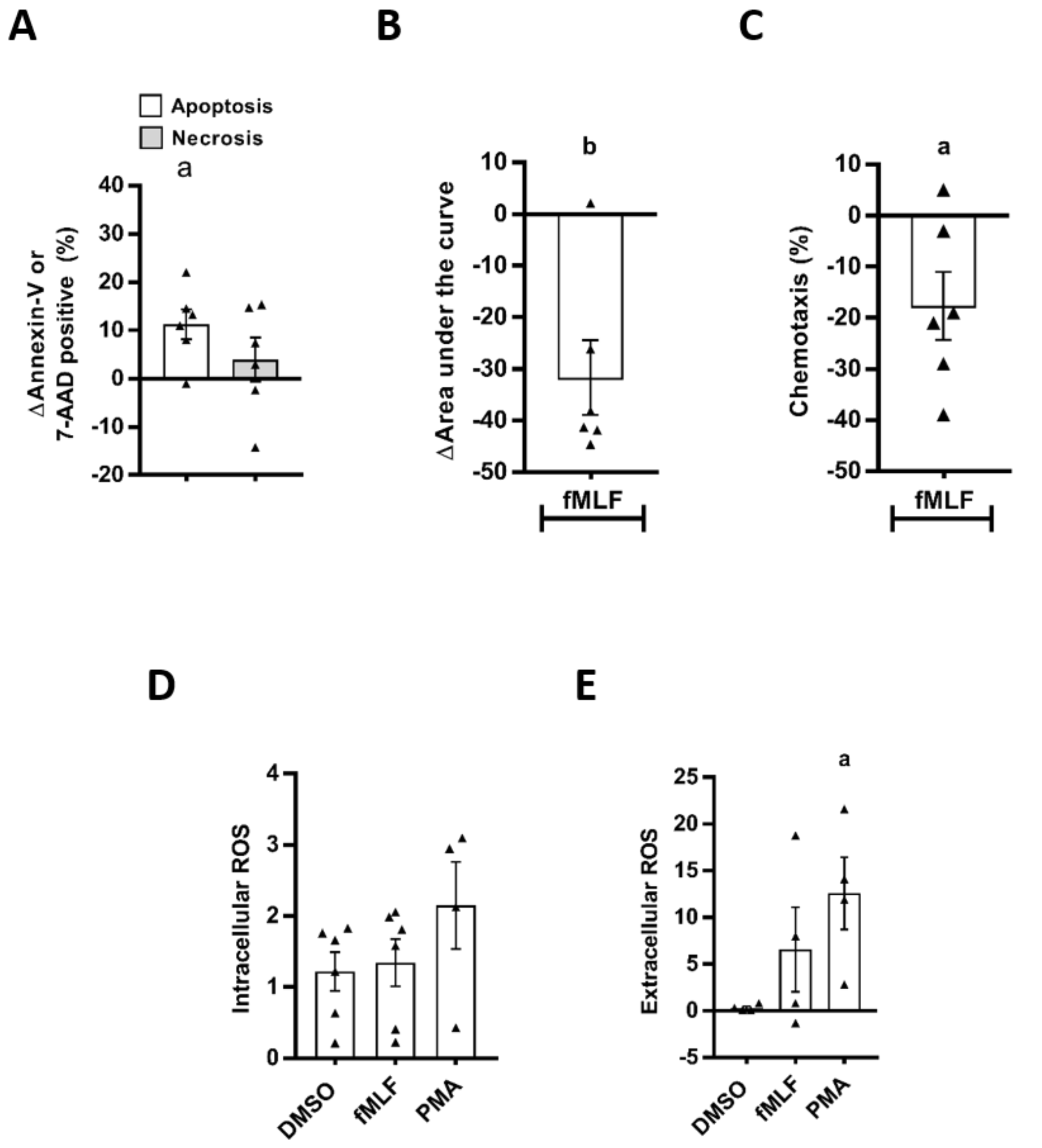

Fig S3: Effect of 48 hours of storage on prednisone-mobilized neutrophil viability and anti-microbial functions. *(A)* Neutrophil mortality expressed as the difference in the proportion of apoptotic and necrotic neutrophils between D1 (day of leukapheresis) and D3 (48 hrs post-leukapheresis) as determined by Annexin-V/7-AAD staining (n=6). *(B)* Change in the fMLF-induced intracellular calcium expressed as the difference in the area under the curve between D1 and D3 (n=6). *(C)* Chemotaxis towards fMLF expressed as the difference in the maximal proportion of migrated neutrophils on D3 and D1 (n=6). *(D)* Intracellular ROS production induced by fMLF and PMA expressed as a ratio of the MFI on D1/D3 (n=6 and n=4, respectively). *(E)* Superoxide anion production induced by fMLF and PMA expressed as the difference in superoxide (mmol/ml) produced on D1

and D3 (n=4). Statistical analysis: One sample t test with a hypothetical mean of 0 (*(A)* to *(C)* and *(E)*) or 1 (*(D)*): [a]p-value < 0.05. [b]p-value < 0.01**.**

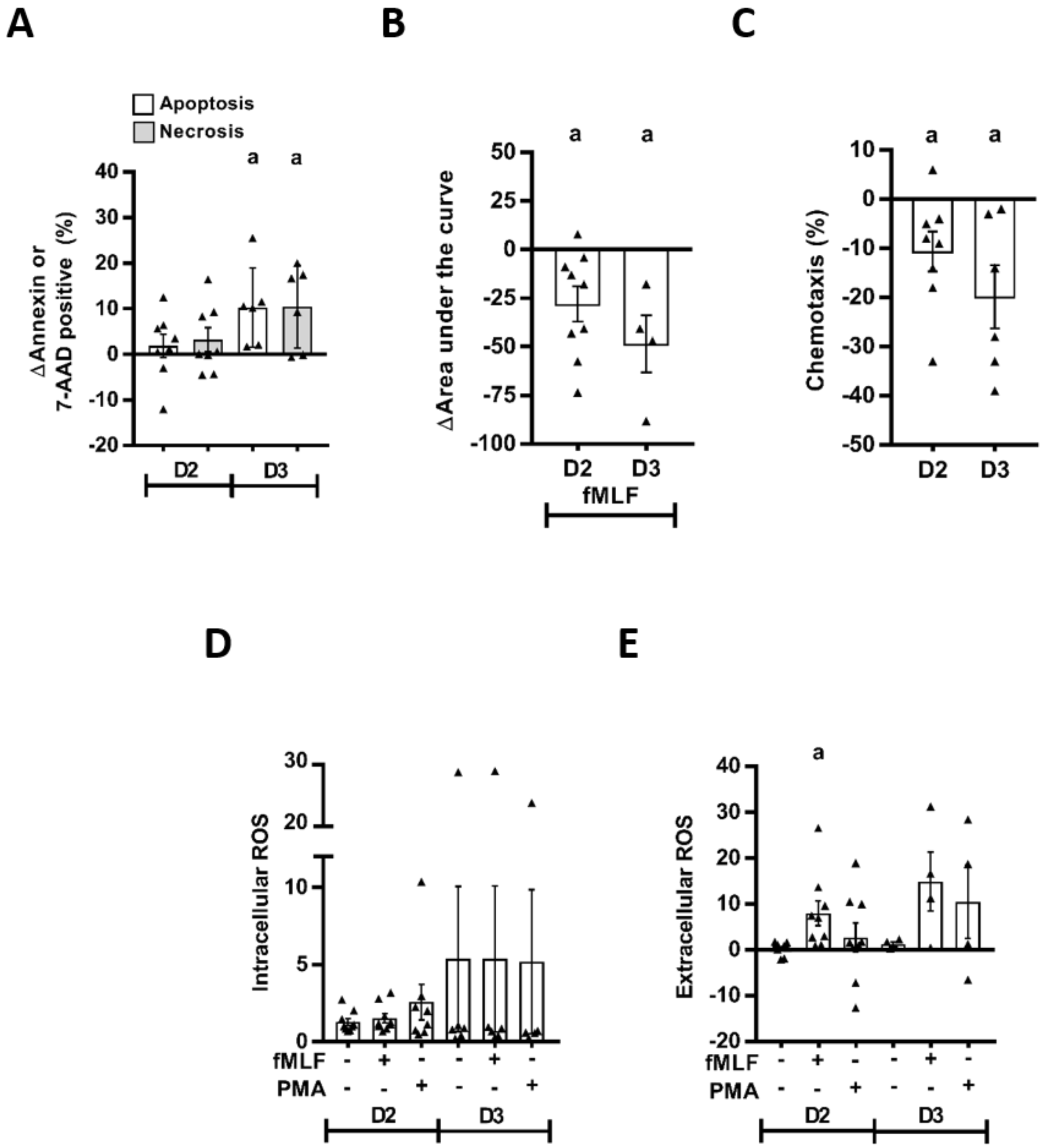

Fig S4: Effect of storage on G-CSF GC neutrophil viability and anti-microbial functions. Viability and functional assay data in *(A)* to *(C)* and in *(E)* are presented as the difference between the values obtained on the day of leukapheresis (D1) and 24 hrs (D2; (D2-D1)) or 48 hrs (D3; (D3-D1)) post-leukapheresis. *(A)* Effect of storage on neutrophil apoptosis *(white bars)* and necrosis *(grey bars)* during storage as determined by Annexin-V/7-AAD staining (D1, D2 (n=8), D3 (n=6)). *(B)* Change in fMLF-induced increase in intracellular calcium expressed as the difference in the area under the curve (D1, D2 (n=9), D3 (n=4)). *(C)* Difference in the maximal proportion of neutrophils that migrated across a chemotaxis chamber towards fMLF (D2 (n=8), D3 (n=6)). *(D)* Neutrophil fMLF and PMA-induced intracellular ROS production expressed as a ratio of the MFI (D1/D2 (n=7) and D1/D3 (n=5)). *(E)* Change in fMLF and PMA-induced superoxide anion production expressed as the difference in mmol/ml of superoxide produced between D1 and D2 (n=9), D3 (n=4).

Statistical analysis: One sample t test with a hypothetical mean of 0 for *(A)* to *(C)* and *(E)*, or 1 for *(D)*: [a]p-value < 0.05.

Supplementary Methods

Gases and metabolite analysis

Gases and metabolites were measured in an undiluted sample of GC with an ABL90 flex blood gas analyser (Radiometer, ON, Canada).

Flow cytometry analysis

Cells were stained with antibodies presented in Table 1 in Supplementary Material at room temperature in the dark for 20 min, washed and resuspended in HBSS 0.5% BSA prior to flow cytometry. The specificity of this antibody panel was confirmed with a Fluorescence Minus One (FMO) control for each fluorochrome and compensation was regularly performed with UltraComp eBeads^{TM} (BD) and Amine Reactive Compensation Bead Kit (ThermoFisher) for FVS605. Ultra Rainbow calibration particles were used for routine calibration control. Data were acquired using a BD LSRII flow cytometer and analysed with Diva software.

Supplementary Table I. Leukocyte Antibody Panel

Antibody	Fluorochrome	Clone	Company	Cat. Number	Target cells
Fixable viability staining	FVS620	NA	Becton Dickinson	564996	Dead cells
CD45	APC-H7	2D1	Becton Dickinson	560274	Leukocytes
CD3	FITC	UCHT1	Becton Dickinson	561806	T cells
CD19	FITC	SJ25C1	Becton Dickinson	340409	B cells
CD56	BV450	NCAM16.2	Becton Dickinson	562752	NK cells
CD14	BV605	M5E2	Becton Dickinson	564055	Monocytes
CD66b	PerCP-Cy5.5	G10F5	Becton Dickinson	562254	Granulocytes
CD193 (CCR3)	PE	5E8	Becton Dickinson	561746	Eosinophils
CD16	BUV395	3G8	Becton Dickinson	563784	Neutrophils
CD15	V500	HI98	Becton Dickinson	561585	Neutrophils
CD10	PeCy7	HI10a	Becton Dickinson	565282	Mature neutrophils
CLEC12A	APC	50C1	BioLegend	353605	Myeloid cells

Flow cytometry gating strategy

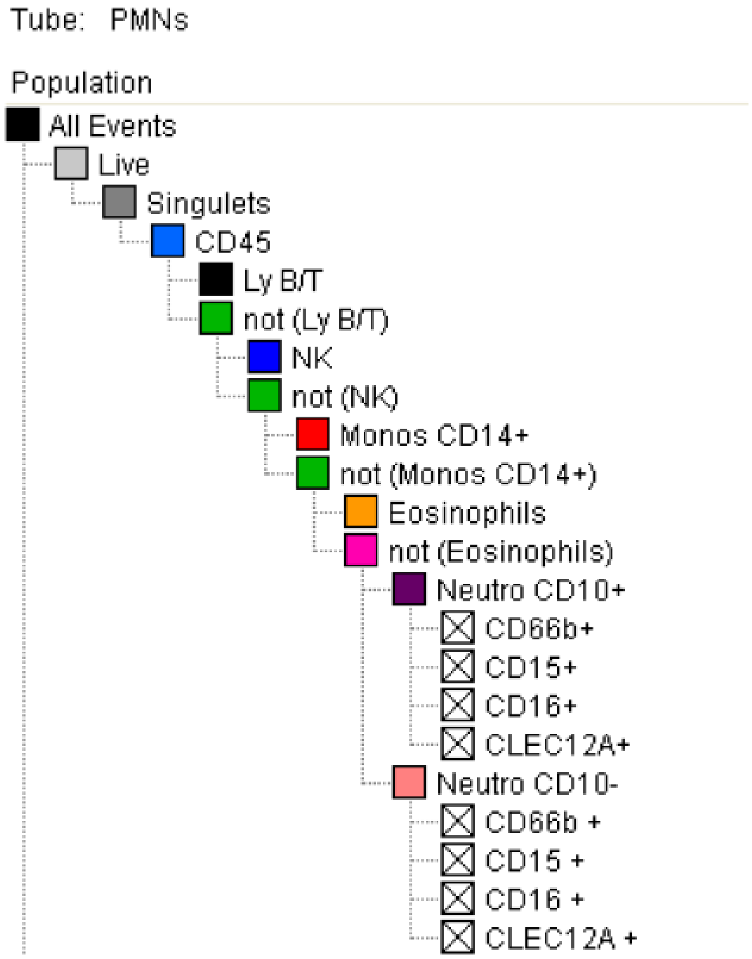

Legend:

Leukocytes = $CD45^+$

Lymphocytes B and T = $CD19^+$ and $CD3^+$

NK cells = $CD56^+$

Monocytes = $CD14^+$

Eosinophils = $CD193^+$

Mature neutrophils = $CD10^+$

Immature neutrophils = $CD10^-$

Purity of non-stimulated, healthy donor neutrophils isolated by density gradient

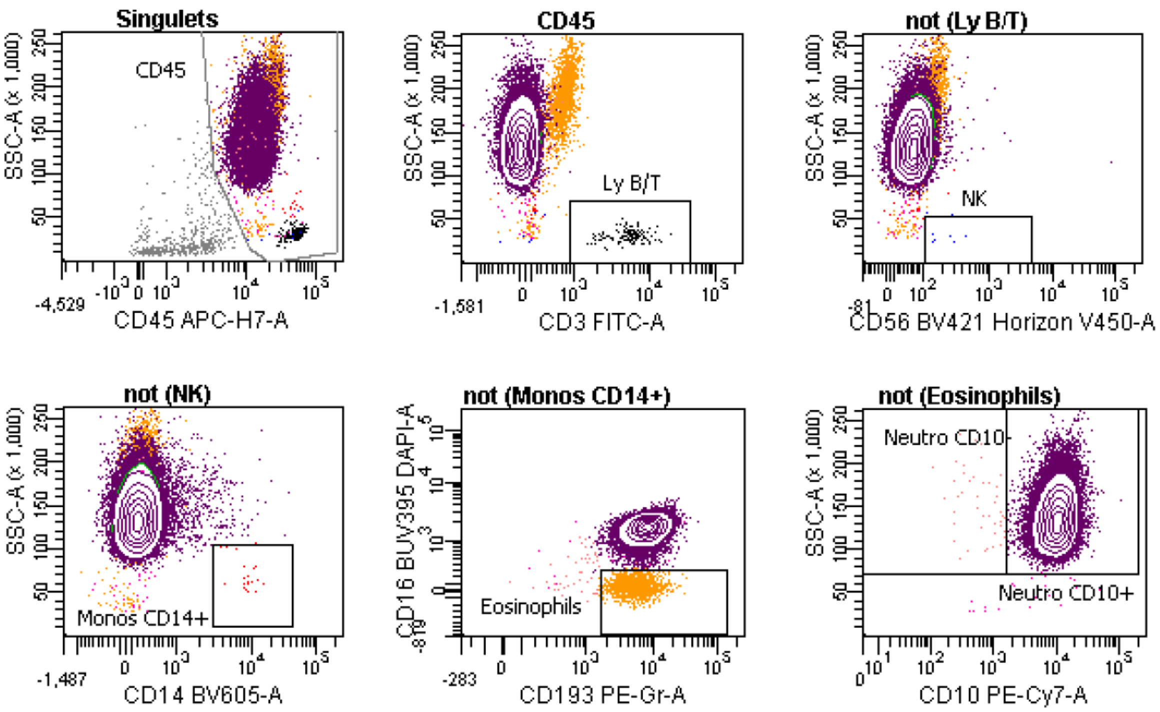

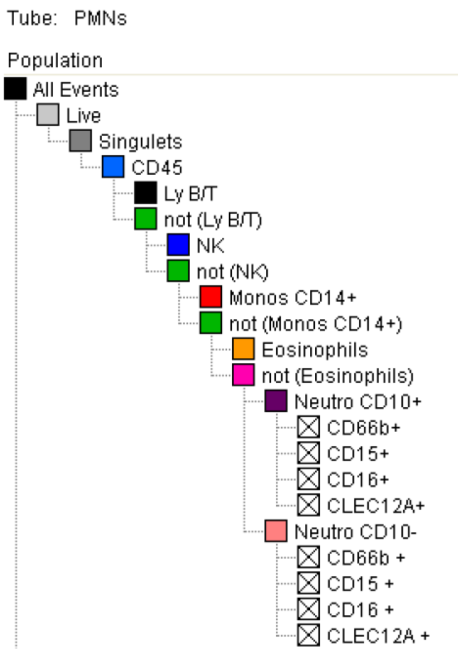

Purity of prednisone GC neutrophils isolated by density gradient

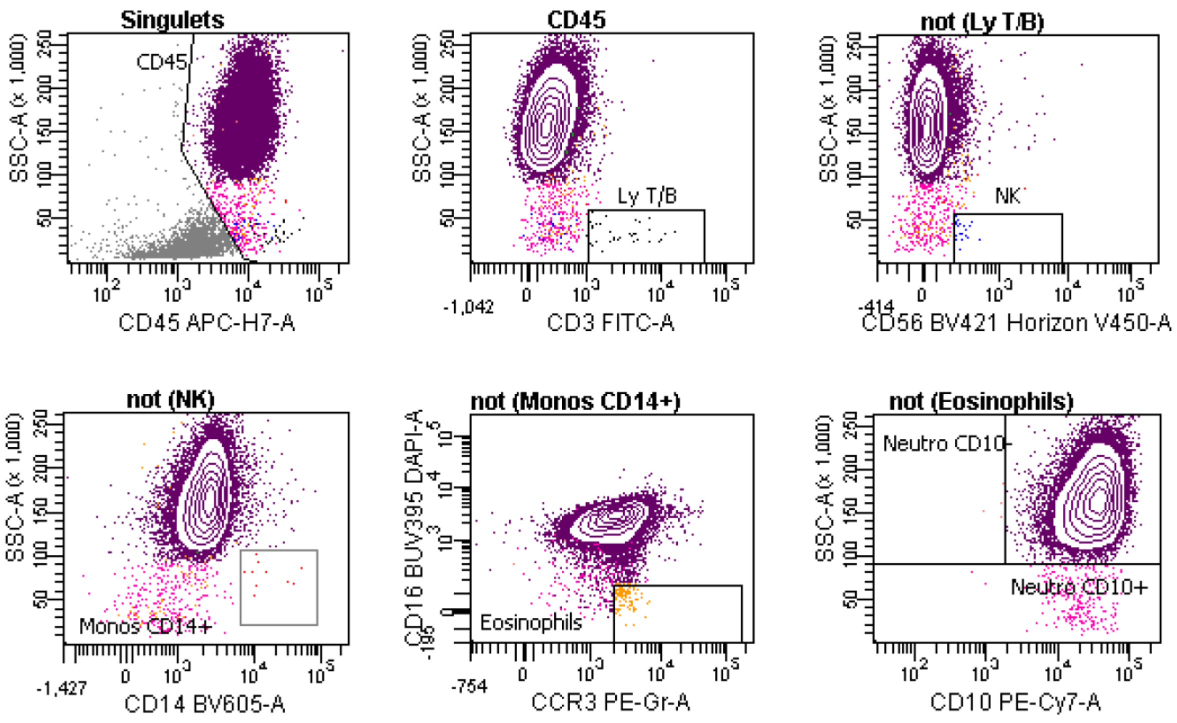

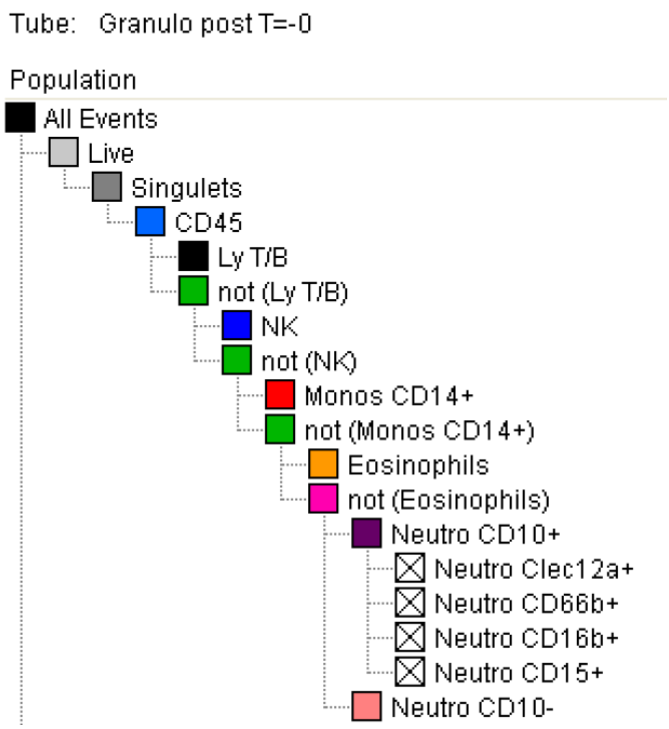

Purity of G-CSF GC neutrophils isolated by density gradient

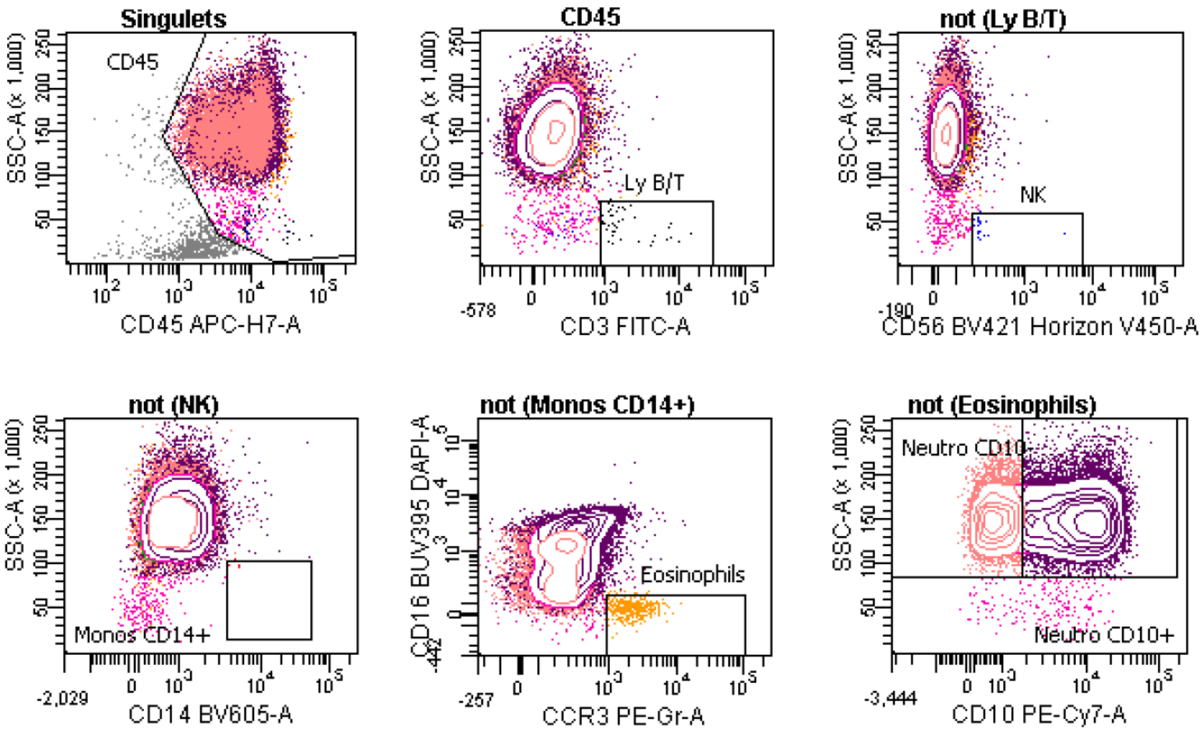

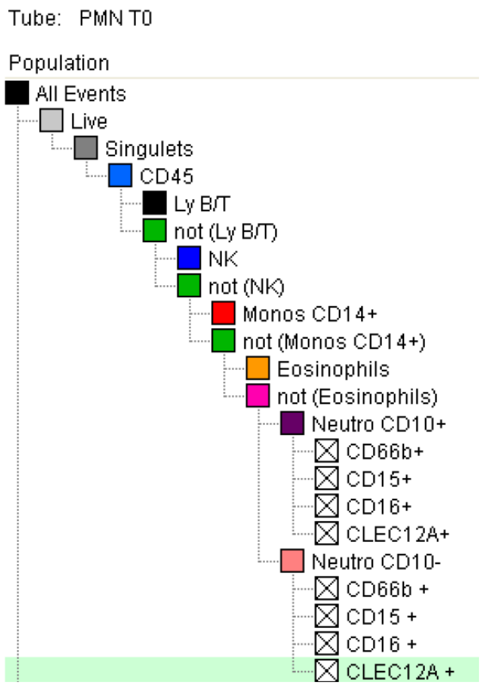

Viability assay

Neutrophils were stained with Annexin-V (BD)/ 7-AAD in Ca^{2+}-free HBSS at room temperature in the dark for 15 min. Cells were kept on ice until analysis with a BD Canto II flow cytometer.

Intracellular Calcium Mobilization

Intracellular calcium concentration after fMLF stimulation was assessed as described in Rousseau et al. (*41*).

Chemotaxis

Chemotaxis towards fMLF was assessed as described in Paré et al (*42*). The results are expressed as the percentage of migrated cells.

Oxidative burst

Neutrophil production of fMLF and PMA-induced intracellular and extracellular ROS was determined as described in Paré et al (*42*).

Phagocytosis

Non-opsonized phagocytosis was quantified after incubating neutrophils with 15µg of pHRodo™ Red zymosan A Bioparticles® Conjugate (Molecular probes, Life technologies). Briefly, cells were centrifuged at 2000 rpm for 15 sec to synchronize phagocytosis prior to a 30 min incubation at 37°C or 4°C for comparative purposes. Phagocytosis was stopped by adding cold HBSS. Cells were kept on ice until analysis with a BD LSRII flow cytometer to determine the proportion of phagocytic neutrophils and the acidification of the phagosomal compartment with the MFI.

Cell staining

Neutrophils and PBMCs were deposited on slides and centrifuged at 3200 rpm for 5 min before staining with Hemacolor® Rapid staining as per the manufacturers' guidelines. Slides were analyzed by microscopy (OLYMPUS BX51) and stored at 4°C.

Cytokine production

Neutrophils were stimulated with 10 $ng.ml^{-1}$ LPS or incubated with the same volume of diluant for 24 hrs at 37°C in RPMI 1640 containing 5 % of decomplemented FBS. Cell-free supernatants were prepared by two successive rounds of centrifugation, the first at 3000 rpm for 3 min and the second at 13 000 rpm for 10 min. Supernatants were stored at -80°C until analyzed by ELISA for IL-8 production with (human IL-8 cytoset, no. CHC1303, ON, Canada) kit as per manufacturer's instruction.

Statistical analysis

Statistical non-parametric analysis was realized with Prism Graph Pad and significance is defined with an alpha of 5%. The prednisone or G-CSF stimulation and the leukapheresis effect were determined with Wilcoxon rank-signed test for paired samples, with Mann-Whitney test when pairing was not possible, or with 1-way ANOVA with Kruskal-Wallis test multiple comparison of prednisone and G-CSF compared with control group. For ROS and IL-8 production analysis, we used 2-way ANOVA with Dunnett's multiple comparisons test with non-stimulated group as reference. The data at D2 and D3 compared to D1 were analyzed with one-sample t-test using a theoretical mean.
